# STRA6 exerts oncogenic role in gastric tumorigenesis by acting as a crucial target of miR-873

**DOI:** 10.1186/s13046-019-1450-2

**Published:** 2019-11-06

**Authors:** Linling Lin, Jian Xiao, Liang Shi, Wangwang Chen, Yugang Ge, Mingkun Jiang, Zengliang Li, Hao Fan, Li Yang, Zekuan Xu

**Affiliations:** 1 Department of General Surgery, The First Affiliated Hospital of Nanjing Medical University, Nanjing, 210000 Jiangsu Province China; 2 Department of General Surgery, Liyang People’s Hospital, Liyang Branch Hospital of Jiangsu Province Hospital, Liyang, 213300 Jiangsu Province China; 3 Department of General Surgery, The First Affiliated Hospital of Nanjing Medical University, 300 Guangzhou Road, Nanjing, 210029 Jiangsu China

**Keywords:** STRA6, Gastric cancer, Proliferation, Metastasis, miR-873

## Abstract

**Background:**

Increasing evidence shows that stimulated by retinoic acid 6 (STRA6) participates in regulating multiple cancers. However, the biological roles of STRA6 in gastric cancer (GC) remain unknown. This study aimed to investigate the biological function of STRA6 and reveal the underlying mechanism of its dysregulation in GC.

**Methods:**

The expression level of STRA6 was detected through quantitative real-time PCR and Western blot analysis. The effects of STRA6 on the proliferation of GC cells were studied through CCK-8 proliferation, colony formation and 5-ethynyl-2′-deoxyuridine (EdU) assays. The effects of STRA6 on migration and invasion were detected via wound healing and Transwell assays. Upstream miRNAs, which might regulate STRA6 expression, was predicted through bioinformatics analysis. Their interaction was further confirmed through dual-luciferase reporter assays and rescue experiments.

**Results:**

STRA6 was up-regulated in GC and enhanced the proliferation and metastasis of GC cells in vitro and in vivo. STRA6 knockdown could inhibit the Wnt/β-catenin signalling pathway. STRA6 was confirmed as an miR-873 target, which acted as a tumour suppressor in GC. Rescue assays showed that the repressing effect of miR-873 could be partially reversed by overexpressing STRA6.

**Conclusions:**

STRA6 is down-regulated by miR-873 and plays an oncogenic role by activating Wnt/β-catenin signalling in GC.

## Background

Gastric cancer (GC) remains a prevalent malignancy worldwide. It was responsible for over 1000,000 new cases in 2018 and approximately 783,000 deaths, making it the fifth-most frequently diagnosed cancer and the third leading cause of cancer-related deaths [1]. Despite remarkable progress on surgical techniques and adjuvant therapy, the prognosis of patients with GC remains poor [2]. GC is frequently diagnosed in advanced stages because of the lack of distinct symptoms and unambiguous molecular signatures [3–5]. Hence, the underlying mechanisms of GC progression should be explored, and new diagnostic markers of GC should be discovered.

In silico analysis of public datasets plays an important role in finding molecular signatures of cancer [6, 7]. The Cancer Genome Atlas (TCGA), a public database that has profiled and analysed large numbers of human tumours to discover molecular aberrations at DNA, RNA, protein and epigenetic levels [8, 9]. By screening the TCGA database, we found that the expression of stimulated by retinoic acid 6 (STRA6) is significantly aberrant between GC tissue and normal tissues. STRA6 encodes a cell surface protein, which is widely expressed in adult organs during development [10]. STRA6 mediates cellular retinol uptake and participates in retinyl ester accumulation in embryonic development [11–13]. STRA6 also functions as a cytokine receptor involved in colon carcinogenesis, fibroblast oncogenic transformation and insulin responses [14, 15]. However, the biological function and underlying mechanism of STRA6 in GC have not been reported.

Gene set enrichment analysis (GSEA) has indicated that related genes of Wnt/β-catenin signalling are enriched in STRA6 overexpression. During cancer growth and development, Wnt/β-catenin signalling has emerged as a fundamental growth control pathway [16, 17]. The dysregulation of Wnt/β-catenin signalling is implicated in many forms of human diseases, including GC [18]. However, whether STRA6 promotes GC progression via Wnt/β-catenin signalling remains unknown.

A high STRA6 mRNA expression with a low copy number gain rate implies that post-transcriptional regulation may be a mechanism of STRA6 up-regulation in GC. The regulation of target genes by miRNAs is a common post-transcriptional regulation method [19]. miRNAs are a class of small non-coding RNAs consisting of 18–25 nucleotides, which down-regulate target mRNA expression by binding to 3′-untranslated regions (3′-UTR), thereby suppressing translation or promoting degradation of mRNA [20]. Bioinformatics analysis and functional assay have shown that STRA6 is regulated by miR-873.

In this study, we aimed to investigate the functional role of STRA6 in GC and uncover the mechanism by which STRA6 promoted GC progression. We showed that STRA6 had an oncogenic function through Wnt/β-catenin signalling and was negatively regulated by miR-873. Our findings might provide insights into potential treatment strategies for GC.

## Methods

### Public data analysis

A TCGA dataset containing gene expression data (named TCGA-STAD.htseq_fpkm-uq.tsv) was downloaded from the UCSC cancer browser (https://xenabrowser.net/datapages/). In addition, the GSE51575 dataset was obtained from NCBI GEO (https://www.ncbi.nlm.nih.gov/geo/) and the normalized data were extracted from “MINiML formatted family file”.

### Human tissue samples

Human GC tissue and paired adjacent normal tissues were obtained from 80 patients who were diagnosed with GC on the basis of histopathological evaluation and underwent surgery at the Department of General Surgery, The First Affiliated Hospital of Nanjing Medical University, China. All of the collected tissue samples were immediately frozen in liquid nitrogen and stored at − 80 °C until they were required.

### Cell culture

Four human GC cell lines (BGC823, SGC7901, MKN45 and MGC803) and one human normal gastric mucous epithelium cell line (GES-1) were purchased from the American Type Culture Collection (Manassas, VA, USA). The GC cells were cultured in RPMI 1640 medium (Invitrogen) containing 10% fetal bovine serum (FBS, WISENT, Canada) and 1% antibiotics (100 U/ml penicillin G and 100 mg/ml streptomycin) at 37 °C with 5% CO_2_.

### RNA extraction and quantitative real-time PCR

Total RNA extraction and quantitative real-time PCR (qRT-PCR) were conducted as described previously [21]. Results were normalised to β-actin expression. The following primers were used in this study: STRA6 forward, 5′-AGACCAGGTCCCACACTGA-3′ and STRA6 reverse, 5′-TTCATAATAGCCAAAGGCATAAAA-3′; β-actin forward, 5′-GCATCGTCACCAACTGGGAC-3′ and β-actin reverse, 5′-ACCTGG CCGTCAGGCAGCTC-3′; miR-873 forward, 5′-GCAGGAACTTGTGAGTCTCCT-3′; miR-874 forward, 5′-CTGCCCTGGCCCGAGGGACCGA-3′; miR-149 forward, 5′-TCTGGCTCCGTGTCTTCACTCCC-3′; Universal, 5′-GCGAGCACAGAATTAATACGAC-3′and U6 forward, 5′-CTCGCTTCGGCAGCACA-3′ and U6, reverse: 5′-AACGCTTCACGAATTTGCGT-3′. The relative expression level of miRNAs was normalised with snRNA U6. All of the procedures were carried out in triplicate, and relative expression was calculated using the 2-ΔΔCT method.

### Western blot analysis

Total protein was extracted from GC cell lines and paired primary tissues by using RIPA lysis buffer (Beyotime, Shanghai, China). Nuclear protein was extracted with Nuclear Extraction Reagents (Beyotime, Shanghai, China). The isolated proteins were separated through sodium dodecyl SDS-PAGE and transferred to a PVDF membrane. Afterwards, the membranes were blocked with 5% non-fat milk at room temperature (RT) for 2 h and incubated with the specific primary antibodies at 4 °C overnight. TBST was used to wash the membranes before and after the membranes were incubated with secondary antibodies. The relative expression levels of the proteins were detected using an ECL detection system. GAPDH was used as an internal control.

### Lentivirus construction and cell transfection

Commercially available lentiviral vectors containing STRA6- and shRNA-coding sequence (STRA6 and sh-STRA6) were constructed by Genechem (Shanghai, China) to up- or down-regulate STRA6 in GC cells. The scrambled lentiviral construct was set as a negative control, and all of the vectors were verified by DNA sequencing. When the GC cell lines MGC03 and SGC7901 grew to 30 to 40% confluency, they were infected with STRA6, vector, sh-STRA6 and sh-NC at a suitable multiplicity of infection. Stable cell lines were obtained using 5 μg/ml puromycin (Sigma, Aldrich) for approximately 1 week. The STRA6 expression of the cells was analysed through qRT-PCR and Western blot analysis. miR-873-mimics, miR-873-inhibitor, miR-874-mimics and miR-149-mimics were transfected using a Lipofectamine 3000 transfection reagent (Invitrogen). miR-NC was used as a negative control.

### Cell proliferation assay

Cell proliferation was analysed with Cell Counting Kit-8 (CCK-8) (Beyotime, Shanghai, China) in accordance with the manufacturer’s recommendations. The cells were plated in 96-well plates (1000 cells/well) and cultured in RPMI 1640 containing 10% FBS for 5 days. In this experiment, 10 μl of CCK-8 reagent was added to each well and incubated at 37 °C for 2 h. Absorbance was detected spectrophotometrically at 450 nm. Each group was analysed three times.

### Colony formation assay

A total of 500 stable transfected GC cells were plated in a six-well plate and maintained in RMPI-1640 medium containing 10% FBS for approximately 2 weeks. Proliferating colonies were fixed with methanol and stained with 1% crystal violet (Beyotime, Shanghai, China). The colonies were counted and photographed. Each group was analysed three times.

### 5-Ethynyl-2′-deoxyuridine (EdU) assay

Cell proliferation was measured via the EdU assay. The cells were cultured in 96-well plates (5 × 10^3^ cells/well) with RPMI 1640 (10% FBS) for 24 h, incubated with 50 μM EdU at 37 °C for 2 h, treated with 4% paraformaldehyde and 0.5% Triton X-100 and stained with 1× Apollo® reaction cocktail for 30 min. Lastly, nuclei were stained with 1 × Hoechst33342, and the cells were visualised under a fluorescence microscope (Nikon, Japan).

### Cell migration and invasion assays

A 6.5 mm chamber with 8 μm pores (Corning Costar Corp., USA) was used to assess the migratory and invasive abilities of GC cells. In this experiment, 2 × 10^4^ transfected cells were plated in the upper chamber with 200 μl of serum-free RMPI-1640 medium. Next, 500 μl of the medium with 10% FBS was added to the lower chamber as a chemoattractant. After 24 h of incubation, the cells that migrated to the lower surface of the filter were stained with 1% crystal violet for 30 min. For invasion assays, 0.1 ml of Matrigel (50 μg/ml, BD Biosciences, USA) was added onto the upper chamber before the cells were plated, and the remaining operating steps were similar to the previous steps. The experiments were performed in triplicate.

### Flow cytometric analysis

The transfected cells were collected, washed carefully with phosphate-buffered saline (PBS), fixed with 75% ethanol, stored at − 20 °C overnight, incubated with RNAse and stained with propidium iodide staining solution (MultiSciences, Hangzhou, China) at RT for 15 min for the cell cycle analysis.

The apoptotic assay was conducted using an Annexin V-APC/PI Apoptosis Detection Kit (Multisciences, Hangzhou, China) and analyzed with a flow cytometry (FACScan, BD Biosciences). The ratio of early and terminal apoptotic cells was detected to calculate the apoptotic rate.

### TOP-flash/FOP-flash luciferase reporter assay

Cells in each group were co-transfected with TOP flash or FOP flash expression plasmid and pRL-TK using Lipofectamine 3000. Luciferase activity was determined with the Dual Luciferase Reporter Assay (Promega). Firefly luciferase activity was normalized to Renilla luciferase activity, and the results were represented as normalized TOP/FOP ratio.

### Immunofluorescence analysis

Stably transfected cells were washed with cold PBS, fixed with 4% PFA for 15 min, washed thrice with PBS and permeabilised with 0.5% Triton X-100 (PBS) at RT for 10 min. Non-specific binding was blocked with normal goat serum (Invitrogen). Primary antibodies, namely, β-catenin (1:100, proteintech), N-cadherin (1:100, proteintech) and vimentin (1:100, CST), were applied at 4 °C overnight. The cells were washed again and then incubated with anti-rabbit antibody at RT for 1 h. The cell nuclei were stained with DAPI for 5 min. Images were captured with a fluorescence microscope (Nikon, Japan).

### Dual-luciferase reporter assay

The 3′-UTR sequences of STRA6 containing wild-type or mutated miR-873 binding sites were synthesised and cloned into a pGL3 luciferase reporter vector (Promega, USA). The cells in 24-well plates were co-transfected with either miR-873 mimic or miR-NC with pGL3-WT-STRA6 or pGL3-MUT-STRA6 by using Lipofectamine 3000 (Invitrogen). After 48 h of transfection, luciferase assay was performed using a dual-luciferase kit (Promega, USA) in accordance with the manufacturer’s protocol. Relative firefly luciferase activities were normalised to *Renilla* luciferase. The experiment was performed in triplicate.

### Hematoxylin and eosin staining of tissue

Firstly, the tissue samples fixed in alcohol were rehydrated using microscope slides. Then we agitated the slides for 30s in deionized water to hydrate the tissues. The slides were then placed into a bottle filled with hematoxylin, agitated for 30 s and washed in deionized water for 30 s. 1% eosin Y solution was used to stain the slides and 95% alcohol followed by 100% alcohol were used to rehydrated the samples. Finally, we used xylene to extract the alcohol and then covered the slides.

### Animal experiment

For the tumour xenograft model, a total of 20 female nude mice were randomly allocated to four groups (MGC803-sh-NC, MGC803-sh-STRA6, SGC7901-vector and SGC7901-STRA6), stable cells (1 × 106 cells/100 μl of PBS) were injected into the flanks of the nude mice in the respective groups. The tumour volume was measured every 4 days and calculated using the following equation: volume = (length × width2)/2. Lastly, the mice were euthanised after 3 weeks. For the metastasis model, the other 14 mice were randomly divided into two groups: negative control and STRA6 knockdown group (*n* = 7/group), and stable cells with sh-NC and sh-STRA6 (1 × 10^6^ cells/100 μl of PBS) were injected into the tail vein of mice. After 5 weeks, the occurrence of distant metastasis was observed using an IVIS imaging system (Caliper life Sciences, Hopkinton, MA, USA).

### Statistical analysis

Each experiment was repeated independently at least three times. Experimental data were shown as mean ± standard deviation (SD) and statistically analysed using SPSS v19.0. Clinicopathological results were compared using Pearson χ^2^ tests. Student’s t-test and ANOVA were used to compare the treated group and control group. *P* < 0.05 was considered statistically significant.

## Results

### STRA6 is up-regulated in GC

**Fig. 1:**
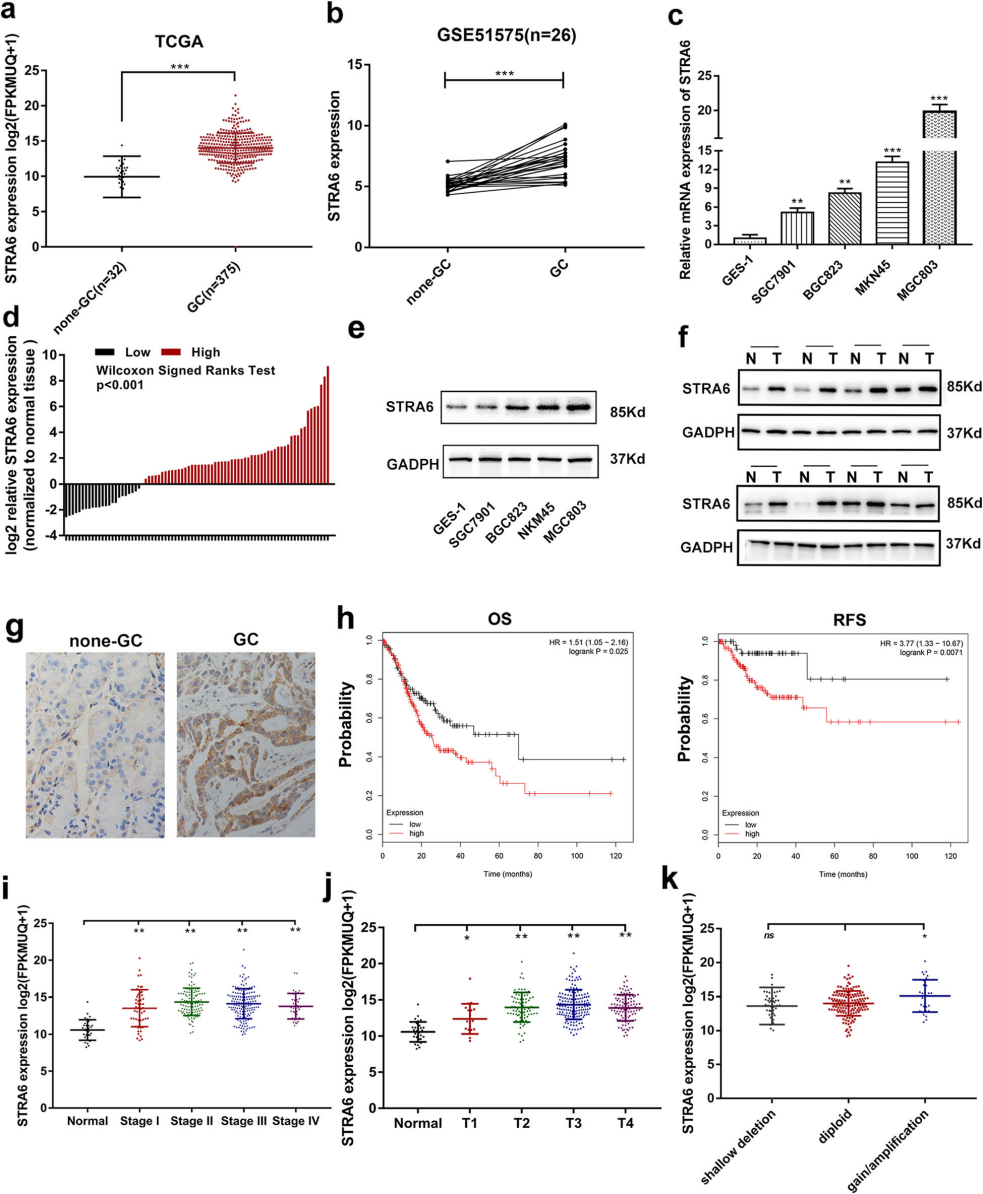
STRA6 is overexpressed in GC and is correlated with poor prognosis with GC patient. **a** and **b** The expression of STRA6 according to TCGA and GEO database. **c** The mRNA expression of STRA6 in GC cell lines and GES-1. **d** The mRNA expression of STRA6 in 80 pairs of GC tissue and adjacent normal tissue. **e** and **f** The protein level of STRA6 in cell lines and tissues. **g** Immunohistochemistry staining was used to determine the protein level of STRA6 in GC tissues and none-GC tissues. **h** High expression of STRA6 was associated with poor overall survival and relapse free survival of GC patients based on TCGA dataset. **i** and **j** Expression patterns of STRA6 based on Stage and T stages of GC. **k** STRA6 mRNA expression patterns within different copy number variants in GC. (*p < 0.05, **p < 0.01, ***p < 0.001. The data expressed as the mean ± SD)

The public dataset (TCGA and NCBI/GEO/GSE57515) showed that the expression level of STRA6 in GC samples was higher than that in the non-GC samples (Fig. 1a and b). The expression levels of STRA6 in 80 pairs of GC and adjacent normal tissues, GC cell lines (MKN45, MGC803, BGC823 and SGC7901) and GES-1 were measured via qRT-PCR. As shown in Fig. 1c and d, the results showed that the STRA6 expression was significantly increased in the GC cell lines and tissues (Fig. 1c and d). The GC cell lines and specimens were used to determine the protein expression level of STRA6 through Western blot analysis. The result showed that the STRA6 expression was higher in the GC cell lines and tissues than in GES-1 and the adjacent non-tumour tissues (Fig. 1e and f, Additional file 1: Figure S1). Moreover, IHC was conducted to further demonstrated the upregulation of STRA6 in GC and the result was consistent with previous findings (Fig. 1g).

### STRA6 overexpression is correlated with the poor prognosis of patients with GC

The Kaplan Meier plot (http://www.kmplot.com) showed that a high STRA6 expression level was correlated with poor overall survival (*P* < 0.025; Fig. 1h, left panel) and relapse-free survival (*P* < 0.0071; Fig. 1h, right panel). The TCGA cohort was analysed to evaluate the correlation between the expression of STRA6 and the clinicopathological features of GC. Advanced stage (Fig. 1i) and T stage (Fig. 1j) were associated with the STRA6 overexpression. The copy number gain rate was positively correlated with the mRNA expression of STRA6 (Fig. 1k). Furthermore, according to STRA6 mRNA expression level, 80 pairs of patients with GC were divided into two groups to investigate the correlation between the STRA6 expression and the clinicopathological features of patients with GC. As shown in Table 1, a high STRA6 expression was correlated with a large tumour size, a late T grade and a poor histological type. However, other factors, such as age and gender, were not correlated with STRA6 abundance.

**Table 1:** Expression of STRA6 in human gastric cancer according to patients’ clinicopathological

Characteristics	Number	STRA6 expression		*P* value
		High group	Low group	
Age (years)				
<60	38	23	15	0.248
≥ 60	42	20	22	
Gender				
Male	46	26	20	0.563
Female	34	17	17	
Size (cm)				
<3	38	15	23	0.015*
≥ 3	42	28	14	
Histological type				
Well-moderately	35	14	21	0.030*
Poorly signet	45	29	16	
Stage				
I/II	38	18	20	0.276
III/IV	42	25	17	
T grade				
T1 + T2	31	11	20	0.009*
T3 + T4	49	32	17	
Lymph node metastasis				
Absent(N0)	32	14	18	0.143
Present (N1 + N2 + N3)	48	29	19	

*p<0.05 statistically significant difference

### STRA6 promotes GC cell proliferation in vitro and in vivo

**Fig. 2:**
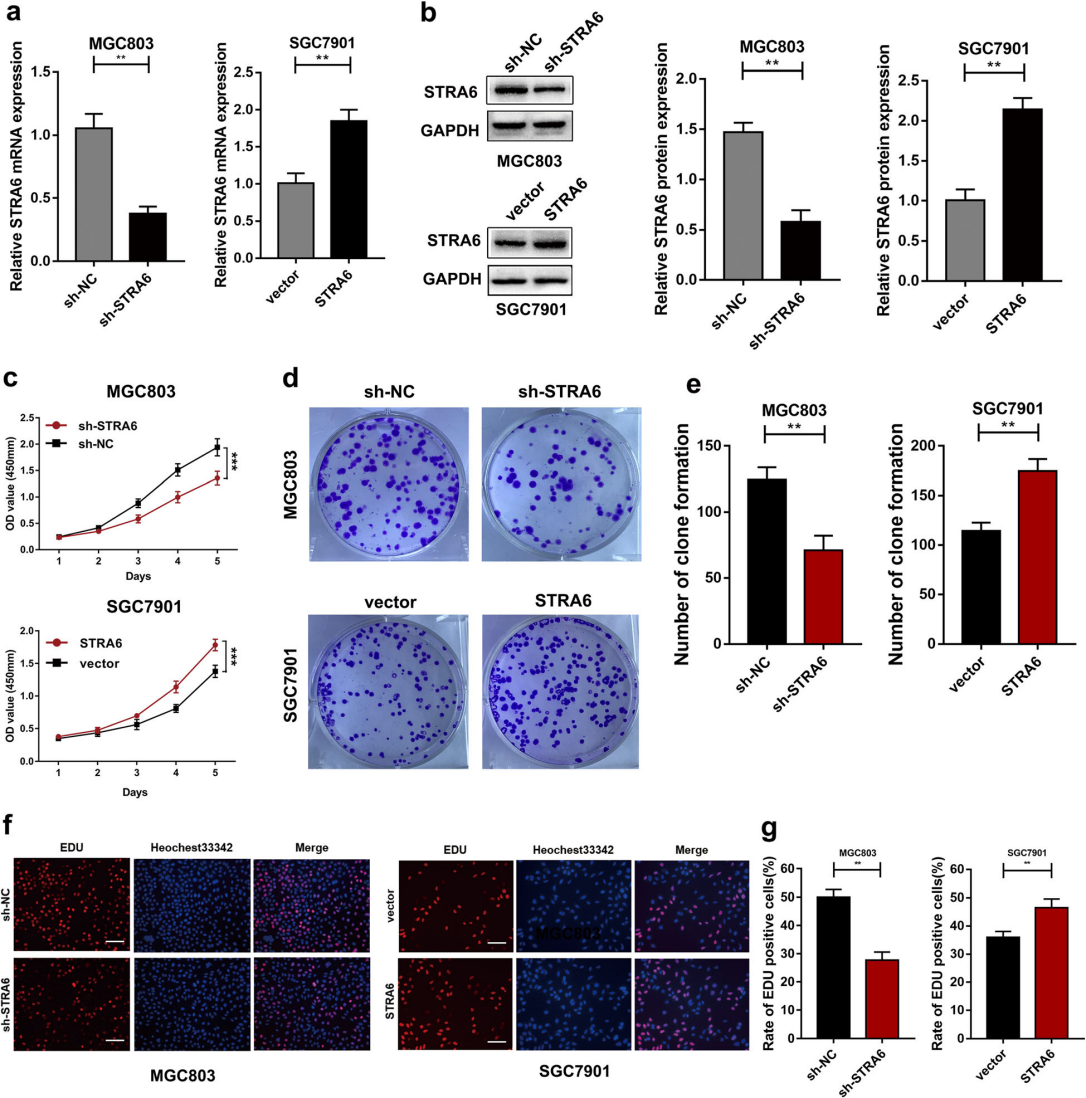
STRA6 facilitate the proliferation of GC. **a** and **b** The transfection efficiency was determined by qRT-PCR and western blot. **c** The effect of STRA6 on proliferation was explored by cck-8 assay. **d** and **e** Colony formation assay was conducted to detect the proliferation abbility of GC cells after transfection. **f** and **g** EdU assay was used to determine the effects of STRA6 on GC cell proliferation. (*p < 0.05, **p < 0.01, ***p < 0.001. The data expressed as the mean ± SD)

**Fig. 3:**
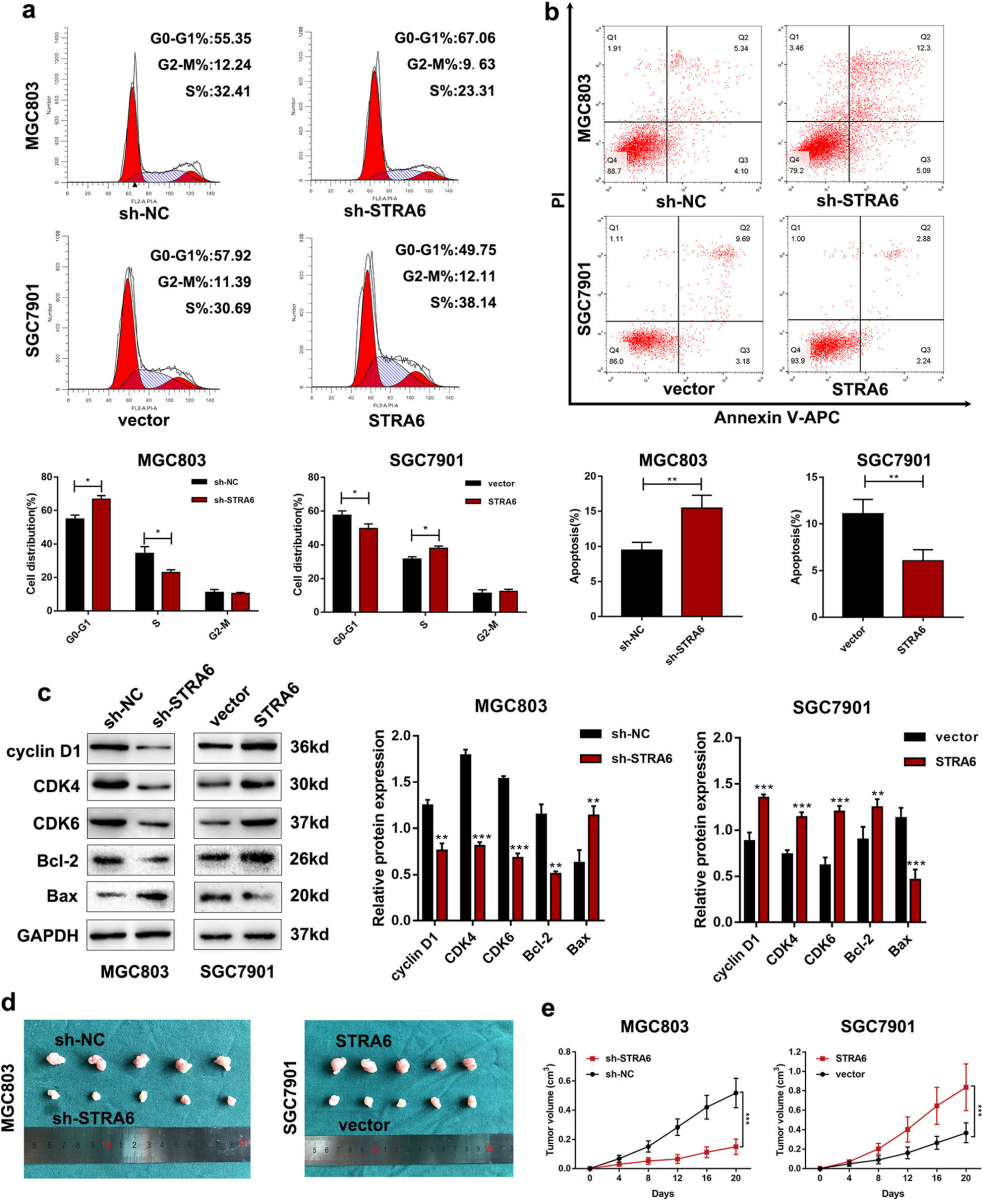
STRA6 knockdown promote cell cycle arrest of GC cell in vitro and facilitate tumorigenicity in vivo. **a** Downregulation of STRA6 promoted G0/G1 cell cycle arrest while ectopic expression of STRA6 presented opposite effect. **b** The apoptotic rates (Q2 + Q3) of transfected cells were detected by flow cytometry. (Q2, early apoptotic cells; Q3, terminal apoptotic cells). **c** Cell cycle-related proteins and apoptosis-related proteins were examined by western blot. **d** and **e** Images of the subcutaneous xenografts from different groups of nude mice transfected with sh-NC, sh-STRA6, vector and STRA6, respectively. Tumour volumes was calculated after injection every 4 days and tumour growth curves were conducted. (*p < 0.05, **p < 0.01, ***p < 0.001. The data expressed as the mean ± SD)

SGC7901 and MGC803 were selected to verify the biological function of STRA6 after the cells were transfected with lentiviral vectors containing STRA6-coding sequence and shRNA-targeting STRA6 (STRA6 and sh-STRA6), respectively (Fig. 2a and b). The growth curves derived from the CCK-8 assay showed that the cell proliferation rate was significantly reduced after the cells were transfected with sh-STRA6; conversely, the cell proliferation rate was increased in response to STRA6 up-regulation (Fig. 2c). Similarly, colony formation assay indicated that the clonality of the GC cells was dramatically suppressed by STRA6 down-regulation but was markedly increased by STRA6 overexpression (Fig. 2d and e). The same results were obtained using EdU proliferation assays (Fig. 2f and g). Flow cytometric assays were performed to further elucidate the mechanism by which STRA6 contributed to cell proliferation. The results showed that the G0/G1 cell cycle arrest was induced and the apoptotic rate was increased in STRA6 down-regulation group (Fig. 3a and b). Likewise, the changes in cell cycle-related proteins (cyclin D1, CDK4 and CDK6) expression and apoptosis-related proteins (Bcl-2, Bax) expression were consistence with the results of flow cytometric analyses (Fig. 3c).

The stably transfected cells were subcutaneously injected into the flanks of nude mice to validate the effects of STRA6 on tumour growth in vivo. The average size of tumours was significantly decreased in the STRA6 knockdown group, and an opposite result was observed in STRA6 overexpression group (Fig. 3d and e).

### STRA6 facilitates GC cell metastasis in vitro and in vivo

**Fig. 4:**
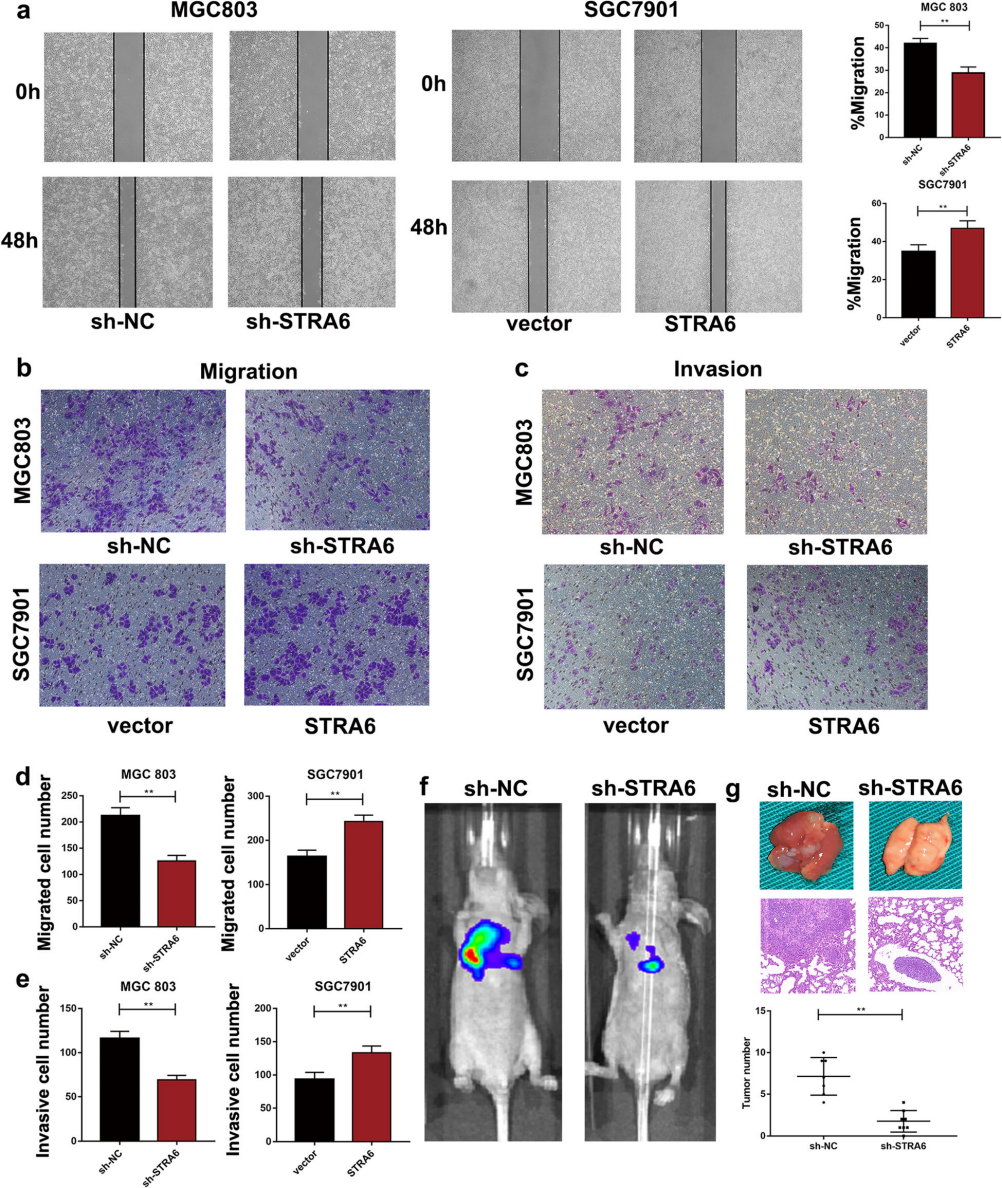
STRA6 facilities migration and invasion of GC in vitro and vivo. **a** Wound healing assay was used to determine the migration the effect of migration of STRA6. **b**-**e** Effects of STRA6 on GC cell migration and invasion were examined by Transwell assay. **f** Representative photographs of tumours were taken by IVIS Imaging System from different groups. **h** Representative pictures of metastatic nodules were presented above. Lung tissues were harvested for H&E staining to characterize the cancerous nodes. And the number of nodules was quantified. (*p < 0.05, **p < 0.01, ***p < 0.001. The data expressed as the mean ± SD)

The relationship between STRA6 expression and GC cell metastasis was investigated. In the wound healing assay, the migration rate was curbed in STRA6 knockdown group but was reinforced in the STRA6 overexpression group (Fig. 4a). In the Transwell assay, after the STRA6 expression was down-regulated, the number of the migrated cells decreased. An opposite effect was detected in the cells subjected to STRA6 overexpression (Fig. 4b and d). Moreover, down-regulating the STRA6 expression decreased the number of invasion cells; by contrast, up-regulating the STRA6 expression reversed the effect (Fig. 4c and e).

The role of STRA6 on tumour metastasis was investigated in vivo. The stably transfected cells were injected into the tail vein of the nude mice, and a bioluminescent signal was assayed 5 weeks after tail vein injection. The result showed that lung metastasis in STRA6 knockdown group was alleviated compared with that of the control group (Fig. 4g and h).

### STRA6 knockdown inhibits the epithelial–mesenchymal transition and Wnt/β-catenin signalling pathway in GC

**Fig. 5:**
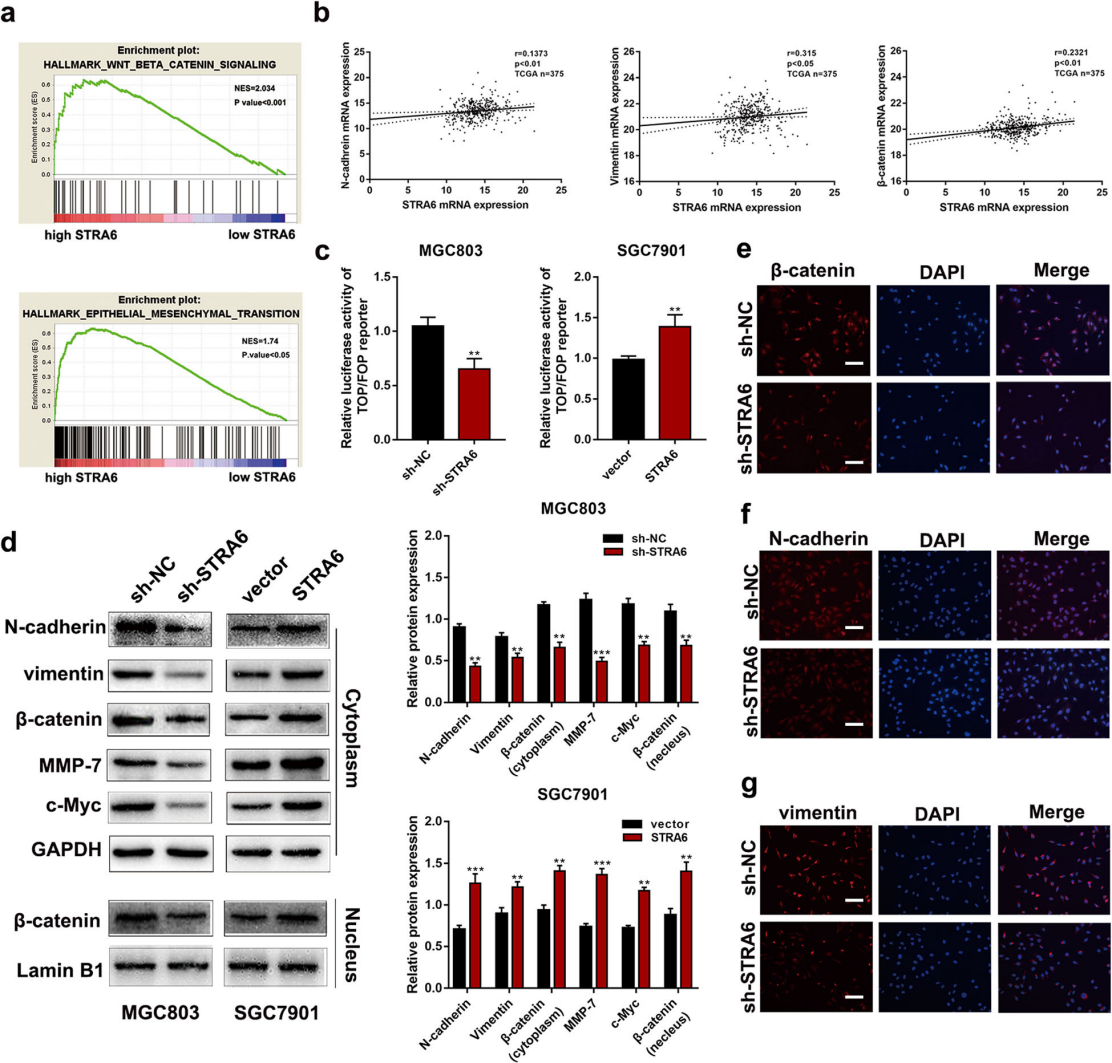
STRA6 knockdown inhibit Wnt/β-catenin signalling pathway and EMT progression. **a** GSEA analysis of STRA6 expression based on TCGA cohort (left panel, *p* value< 0.001; left panel, *p* value< 0.05). **b** The correction of expression between STRA6, β-catenin, N-cadherin and vimentin. **c** The effect of STRA6 on Wnt/β-catenin signalling activity was evaluated by TOP-flash/FOP-flash luciferase reporter assay. **d** Protein level of biomarkers of EMT and Wnt/β-catenin signalling was detected by western blot. **e**-**g** Immunofluorescence staining with β-catenin, N-cadherin, vimentin (Red) and DAPI nuclear staining (blue). (*p < 0.05, **p < 0.01, ***p < 0.001. The data expressed as the mean ± SD)

GSEA via a TCGA cohort indicated that the genes related to Wnt/β-catenin signalling and epithelial–mesenchymal transition (EMT) were remarkably enriched in STRA6 overexpression case (Fig. 5a). To explore the relationship between STRA6 and the two abovementioned pathways, the correlation of the expression between STRA6 and β-catenin and between N-cadherin and vimentin was analysed using the TCGA dataset. As shown in Fig. 5b, the mRNA expression of STRA6 was positively correlated with β-catenin, N-cadherin and vimentin. To further elucidate the role of STRA6 in Wnt/β-catenin signalling, Top/Fop flash luciferase assays were performed. As expected, TOP/FOP transcriptional activity was remarkably inhibited in cells with STRA6-slienced, whereas TOP/FOP activity was increased when STRA6 was up-regulated (Fig. 5c). Western blot analysis further confirmed that STRA6 knockdown significantly decreased the N-cadherin and vimentin expression levels. Moreover, in the STRA6 knockdown group, the expression of MMP-7, c-myc as well as the cytoplasmic and nuclear β-catenin markedly decreased. The STRA6 overexpression showed an opposite effect (Fig. 5d). Immunocytochemistry analysis revealed that STRA6 knockdown inhibited the expression of nuclear β-catenin, N-cadherin and vimentin (Fig. 5e–g).

### STRA6 is negatively regulated by miR-873 in GC

**Fig. 6:**
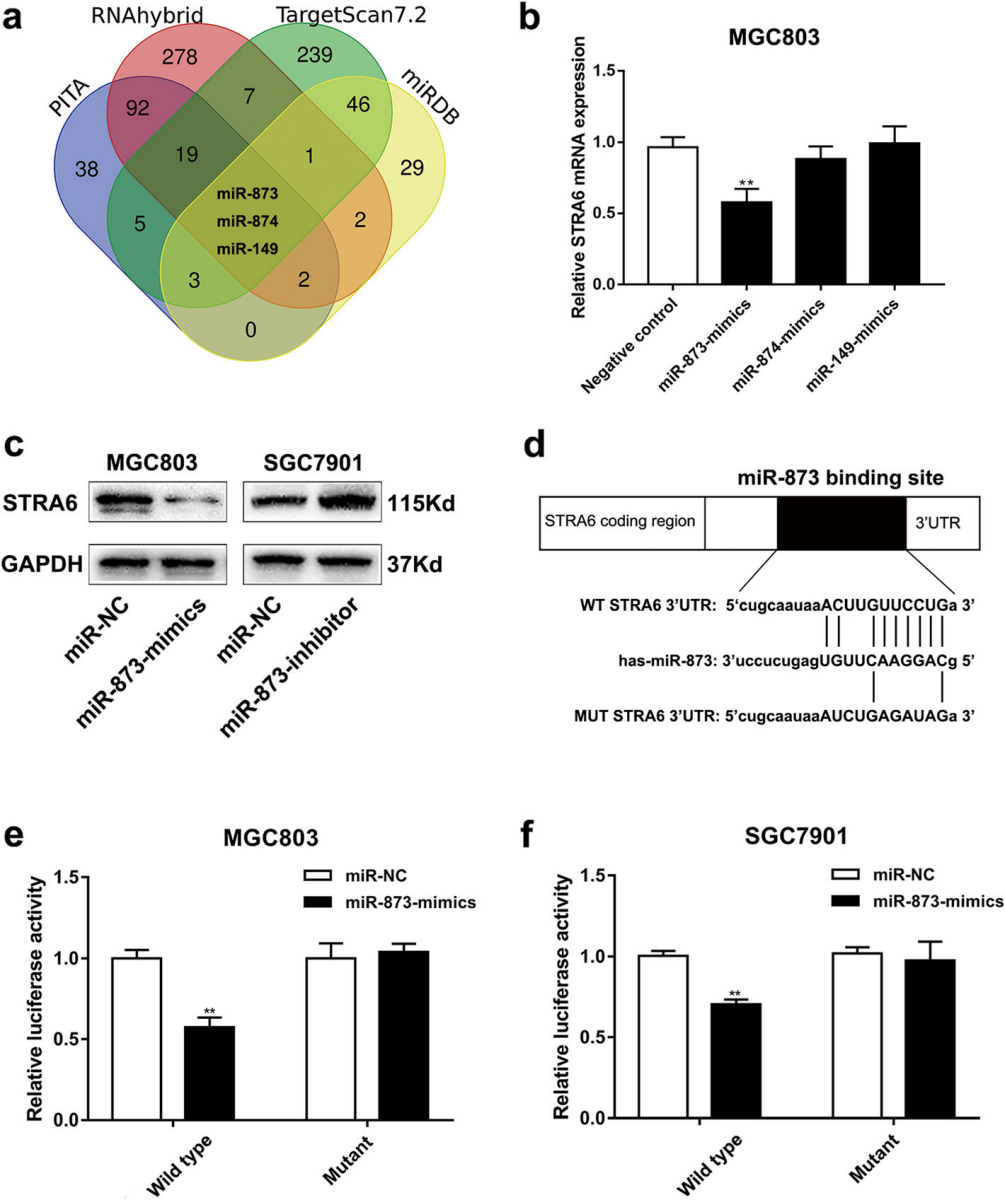
STRA6 is a direct target of miR-873. **a** Putative binding sites in 3′-UTR of STRA6 for the related miRNA binding. **b** The mRNA expression of STRA6 after transfecting with miR-873-mimics, miR-874-mimics and miR-149-mimics in MGC803. **c** The protein level of STRA6 were determined by western blot after transfection. **d** Wild type (WT) and Mutant type (MUT) STRA6 3′UTR sequences were cloned into pGL3 luciferase reporter vector.  **e** and **f **miR-873 inhibited the relative luciferase activity in GC cells co-transfecting with miR-873-mimics and pGL3-STRA6-WT. (*p < 0.05, **p < 0.01, ***p < 0.001. The data expressed as the mean ± SD)

MiRNA dysregulation was investigated to examine the rationale of aberrant STRA6 expression in GC. Three miRNAs that might regulate STRA6 were screened and predicted via four bioinformatics websites (miRDB: https://www.mirdb.org, Targetscans7.2: https://www.targetscan.org/vert_72/, PITA: https://genie.weizmann.ac.il/pubs/mir07/index.html and RNAhybrid: https://omictools.com/rnahybrid-tool, Fig. 6a). To explore whether STRA6 was regulated by miR-873, miR-874 or miR-149, STRA6 expression was detected after up-regulating the candidate miRNAs. The results indicated that only miR-873 reduced the mRNA and protein expression levels of STRA6 (Fig. 6b and c). Dual-luciferase reporter assays were performed to further confirm the direct binding site affinity between STRA6 3′-UTR and miR-873 (Fig. 6d). Notably, in the vector containing the wild-type sequence, the ectopic expression of miR-873 inhibited the luciferase activity in MGC803 and SGC7901 cell lines (Fig. 6e and f). These results revealed that miR-873 regulated the STRA6 expression by directly binding to its 3′-UTR.

### MiR-873 is a tumour-suppressing miRNA down-regulated in GC

**Fig. 7:**
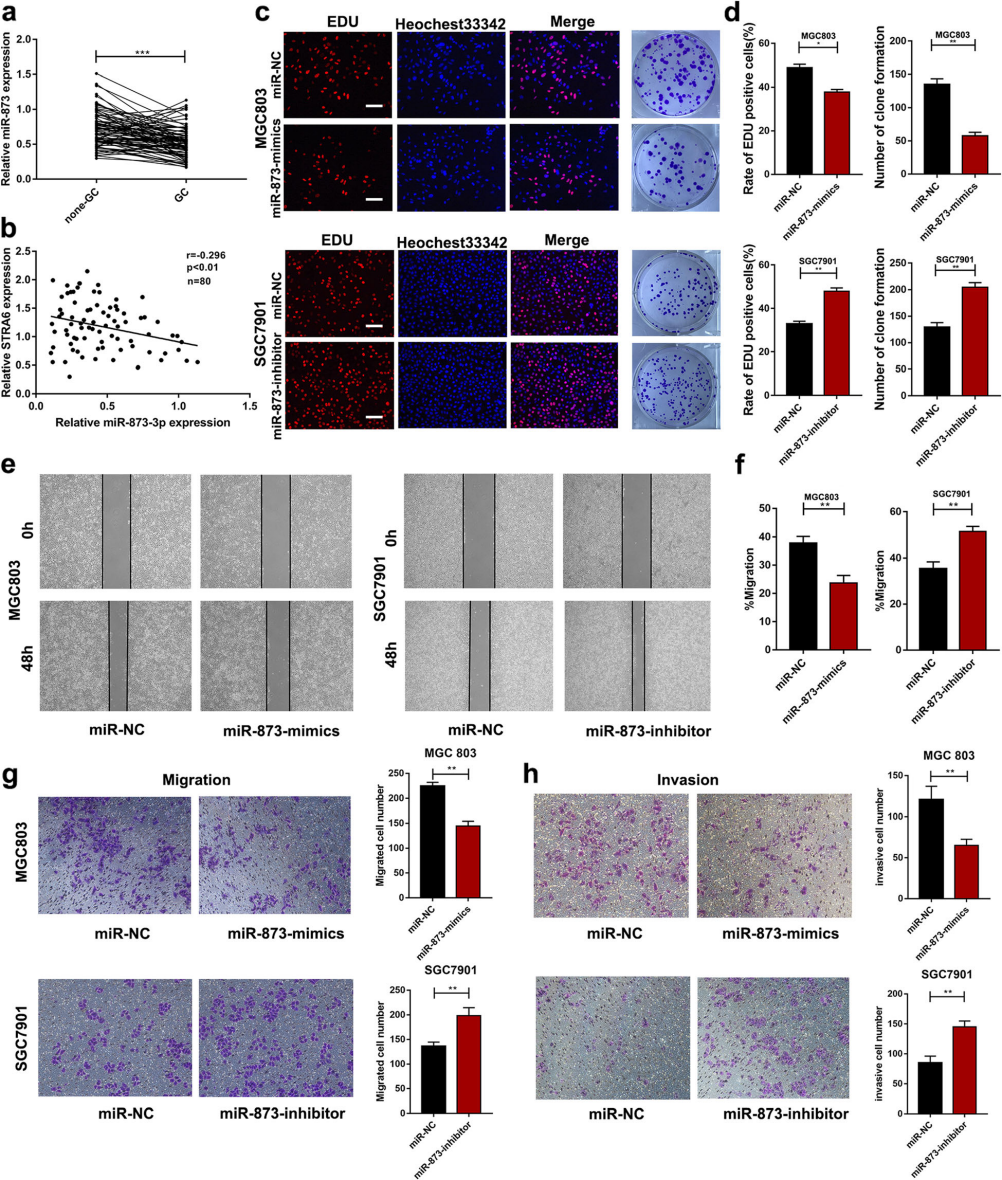
miR-873 is downregulated in GC and play a tumour-suppressive role. **a** miR-873 was downregulated in GC tissues compared to adjacent normal tissues. **b** The correlation of expression between STRA6 and miR-873. **c** and **d** The effect of miR-873 on proliferation in GC cell were detected by Edu assay and colony formation assay. **e** and **f** STRA6 knockdown decreased cell migration ability of GC cells and upregulated expression of STRA6 reversed the effect. **g** and **h** The ability of migration and invasion of transfected cell were determined by Transwell migration and Matrigel invasion assays. (*p < 0.05, **p < 0.01, ***p < 0.001. The data expressed as the mean ± SD)

Given that miR-873 is a regulator of STRA6, we aimed to further explore its biological function. The expression of 80 pairs of GC and adjacent normal specimens was analysed through qRT-PCR, and the results showed that miR-873 was down-regulated in primary tumour tissues (Fig. 7a). Notably, the expression level of miR-873 was negatively associated with STRA6 (Fig. 7b). EdU proliferation assay revealed that the ectopic expression of miR-873 decreased the number of EdU-positive cells, whereas the down-regulated expression of miR-873 reversed the effect. The colony numbers were markedly reduced after the miR-873 expression was up-regulated. This observation was consistent with the result of the EdU assay (Fig. 7c and d). The results of wound healing assay (Fig. 7e and f) and Transwell assay (Fig. 7g and h) suggested that cell migration and invasion abilities were diminished after the miR-873 expression was increased, but both abilities were strengthened after the cells were transfected with miR-873-inhibitor. These results indicated that miR-873 played a tumour-suppressive role in GC.

### STRA6 re-expression rescues the tumour-suppressive function of miR-873

**Fig. 8:**
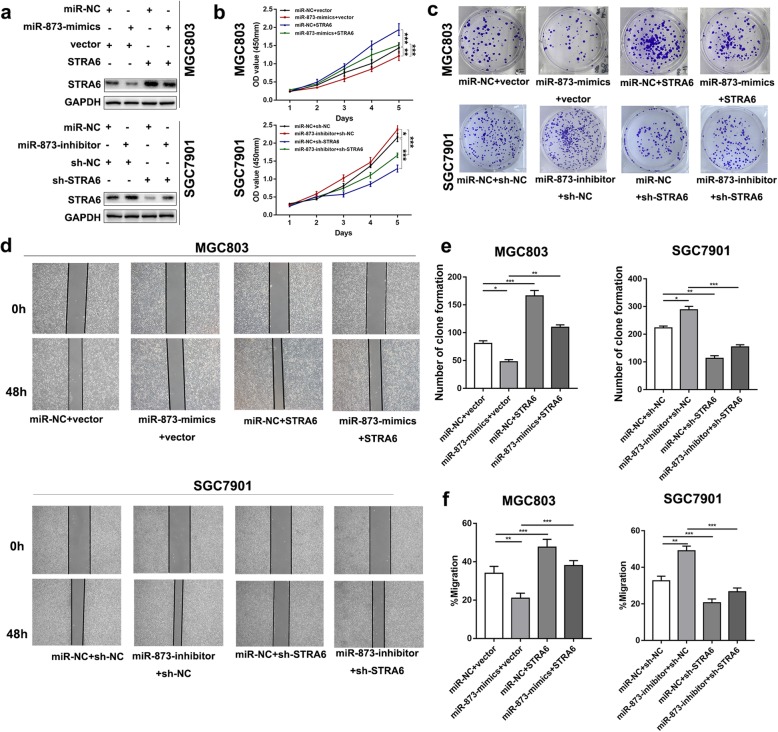
Upregulated expression of STRA6 could partly counteract the effect of miR-873 on GC cells. **a** Efficiency of STRA6 re-expression was detected by Western blot. **b** CCK-8 proliferation assay indicated that repressive effect of miR-873-mimics on cell proliferation could be reversed by overexpression of STRA6. **c** and **e** Partial restoration of clone formation number was observed in the group that co-transfected with miR-873-mimics and STRA6. **d** and **f** Wound healing assay revealed that overexpression of STRA6 partly diminished the tumour-suppressive effect of miR-873. (*p < 0.05, **p < 0.01, ***p < 0.001. The data expressed as the mean ± SD)

Rescue experiments were conducted to investigate whether STRA6 was a function target of miR-873. The STRA6 protein expression in MGC803 was examined via Western blot analysis after the cells were co-transfected with miR-NC + vector, miR-873-mimics+vector, miR-NC + STRA6 and miR-873-mimics+STRA6 respectively (Fig. 8a). CCK-8 (Fig. 8b), EdU (Additional file 2: Figure S2a) and colony formation (Fig. 8c and e) assays indicated that overexpression of STRA6 could reverse the effect of miR-873 on proliferation. Likely, transwell (Additional file 2: Figure S2b) and wound healing assays (Fig. 8d and f) demonstrated that ectopic STRA6 expression partly restored the effects of miR-873 on migration and invasion. Moreover, the results of rescue study in vivo (Additional file 3: Figure S3a and b) were consistence with the proliferation assays and metastasis assays in vitro. These data revealed that upregulation of STRA6 could suppress the effect of miR-873 on cell proliferation and migration in GC.

### miR-873 is involved in Wnt/β-catenin signalling and EMT by targeting STRA6

**Fig. 9:**
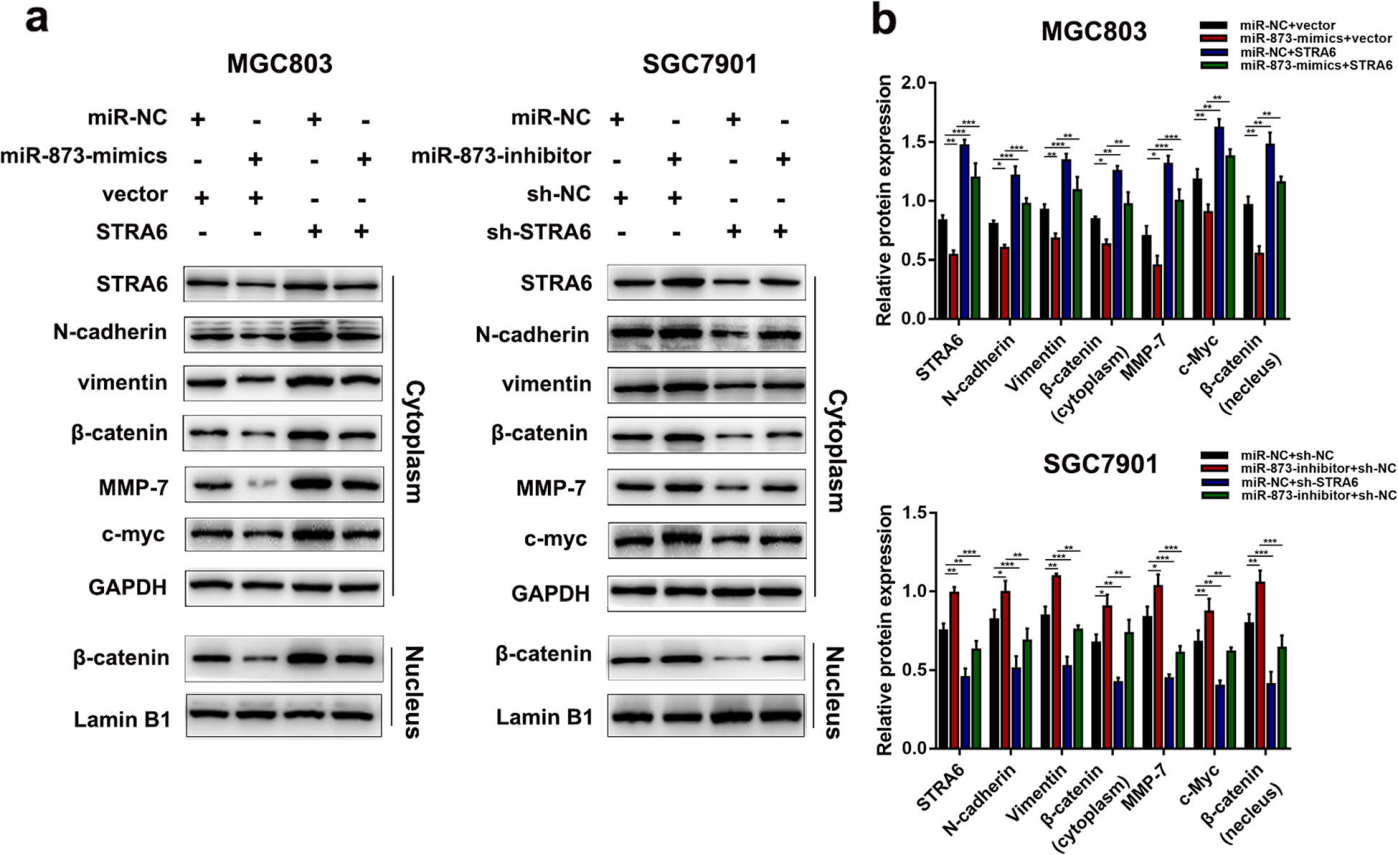
miR-873 is involved in Wnt/β-catenin signalling and EMT in GC cells by targeting STRA6. **a** and **b** Western blot was used to determine the protein expression of genes that related to the pathways after transfecting with miR-873-mimics and miR-873-inhibitor. And protein expression level of these genes was detected after the restoration of STRA6. (*p < 0.05, **p < 0.01, ***p < 0.001. The data expressed as the mean ± SD)

STRA6 knockdown could inhibit Wnt/β-catenin signalling and EMT progression. As such, this study was conducted to determine if these effects could be regulated by miR-873. The result of TOP/FOF flash luciferase assays demonstrated that restoring STRA6 reversed the inhibitory effect of miR-873 on Wnt–β-catenin signalling activation (Additional file 4: Figure S4). Meanwhile, the expression level of EMT-related markers was detected to evaluate the relationship between miR-873, STRA6 and EMT through Western blot analysis. As shown in Fig. 9a and b, the miR-873 overexpression decreased the expression of EMT and Wnt/β-catenin signalling pathway related genes in MGC803, but this change could be mitigated by the STRA6 overexpression. Simultaneously, the expression of genes related to EMT and the Wnt/β-catenin signalling pathway was enhanced after miR-873 was silenced. These effects were partially blocked by down-regulating the STRA6 expression. Therefore, miR-873 was involved in Wnt/β-catenin signalling and EMT progression by targeting STRA6.

## Discussion

STRA6, as a transmembrane protein of RA, is overexpressed in many cancer types [15, 22]. However, the function and potential molecular mechanism of STRA6 up-regulation in GC remain to be elucidated. In this study, qRT-PCR and Western blot analysis revealed that STRA6 was highly expressed in GC cell lines and tissues. A high STRA6 expression level was correlated with a large tumour size, an advanced T grade and a poor histological type. Functional investigations indicated that STRA6 exerted an oncogenic role by promoting the proliferation, migration and invasion of GC cells. These findings showed that STRA6 was up-regulated in GC and had a therapeutic potential for patients with GC.

STRA6 knockdown could inhibit EMT and Wnt/β-catenin signalling in GC. Previous studies confirmed that Wnt/β-catenin signalling is associated with tumour proliferation and metastasis [23, 24]. Wnt ligands bind to Frizzled receptor complexes and activate Frizzled, which stabilises cytoplasmic β-catenin protein by inhibiting the protein destruction complex (APC, axin, GSK3β and CK1). The down-regulation of APC contributes to the nuclear accumulation of β-catenin, which subsequently interacts with a LEF/TCF transcription factor to activate the transcription of target genes [25]. In the present study, the related genes of Wnt signalling, such as β-catenin, MMP-7 and c-myc, were up-regulated because STRA6 was overexpressed, indicating that STRA6 contributed to the activation of the pathway.

EMT is the biological process through which epithelial cells are transformed into specific stromal phenotype cells via specific mehcanisms [26]. Numerous reports have indicated that EMT may be involved in the initial steps of the metastatic cascade, including tumour invasion, intravasation and micrometastasis formation [27]. EMT is typically characterised by a decreased expression of cell adhesion proteins and an increased expression of mesenchymal-associated molecules [28]. In the present study, STRA6 knockdown decreased N-cadherin and vimentin expression, suggesting that STRA6 enhanced EMT. This observation was consistent with the results of migration and invasion assays.

Copy number variation (CNV) is a form of genomic structural variation that results in abnormal gene copy numbers, which may affect the expression of cancer-related genes [29–31]. To investigate whether CNVs were associated with STRA6 up-regulation in GC, we analysed the copy number changes and the mRNA expression of STRA6 in the TCGA cohort. However, the mRNA up-regulation of TRA6 in the GC samples could not be explained by copy number changes. Thus, we focused on miRNA dysregulation, which is a type of post-transcription regulation [32, 33]. After screening databases and performing a series of validation assays, we found that miR-873 negatively regulated the STRA6 expression in GC. miR-873 has been identified as a tumour suppressor in colon cancer, breast cancer and lung cancer [34, 35]. Our data suggested that miR-873 inhibited GC proliferation and metastasis. The miR-873 expression was negatively correlated with STRA6. Moreover, rescue assay validated that miR-873 exerted its tumour suppressive role by targeting STRA6, and our study enriched the target pool for miR-873 in GC. The aberrant regulation of STRA6 by this dysregulated miRNA network is novel.

## Conclusions

Our data revealed that the overexpression of STRA6 was associated with the prognosis of GC and exerted an oncogenic role by activating the Wnt/β-catenin signalling pathway. We demonstrated that STRA6 was up-regulated in GC because of miR-873 dysregulation and CNV. To date, our characterisation of this new miR-873/STRA6 axis helped enhance our understanding of GC progression and likely provided a basis for developing therapeutic targets for GC.

## Supplementary information

**Additional file 1: Figure S1.** The protein level of STRA6 in 80 pairs of human samples.

**Additional file 2: Figure S2.** (a) EdU assays was conducted to examine the proliferation ability after co-transfecting with miR-NC, miR-873-mimics, vector or STRA6. (b) Transwell assay was used to analyze cell migration and invasion ability in each group.

**Additional file 3: Figure S3.** (a, b) The effect of miR-873-mimics on cell proliferation and metastasis were reversed by STRA6 overexpression in vivo.

**Additional file 4: Figure S4.** The TOP/FOP transcriptional activity was enhanced after up-regulating the expression of miR-873 and restoring STRA6 could partly reverse this effect. (TIF 200 kb)

## Data Availability

All data generated or analysed during this study are included in this published article.

## Abbreviations

3′-UTR: 3′-untranslated regions
CCK-8: Cell Counting Kit-8
EdU: 5-Ethynyl-2′-deoxyuridine
EMT: Epithelial–mesenchymal transition
GC: Gastric cancer
GSEA: Gene set enrichment analysis
MMP-7: Matrix metallopeptidase 7
PBS: Phosphate-buffered saline
qRT-PCR: Quantitative real-time PCR
RA: Retinol
STRA6: Stimulated by retinoic acid 6
TCGA: The Cancer Genome Atlas
