# Supplementary figures and images for: STRA6 exerts oncogenic role in gastric tumorigenesis by acting as a crucial target of miR-873
Source: J Exp Clin Cancer Res. 2019 Nov 6;38:452. doi: 10.1186/s13046-019-1450-2 (PMC6836487; doi:10.1186/s13046-019-1450-2)

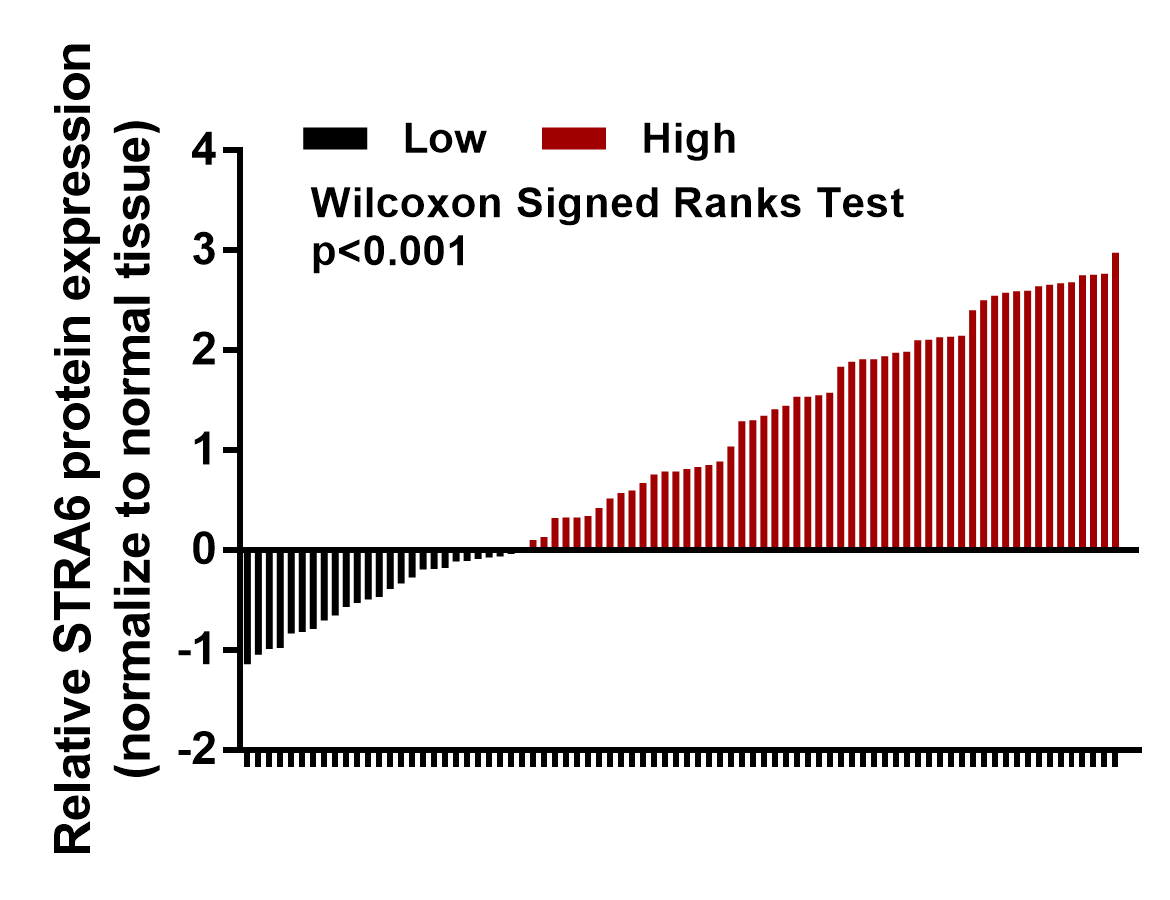

Supplement: Supplementary file 1 — Additional file 1: Figure S1. The protein level of STRA6 in 80 pairs of human samples. [file 13046_2019_1450_MOESM1_ESM.tif]

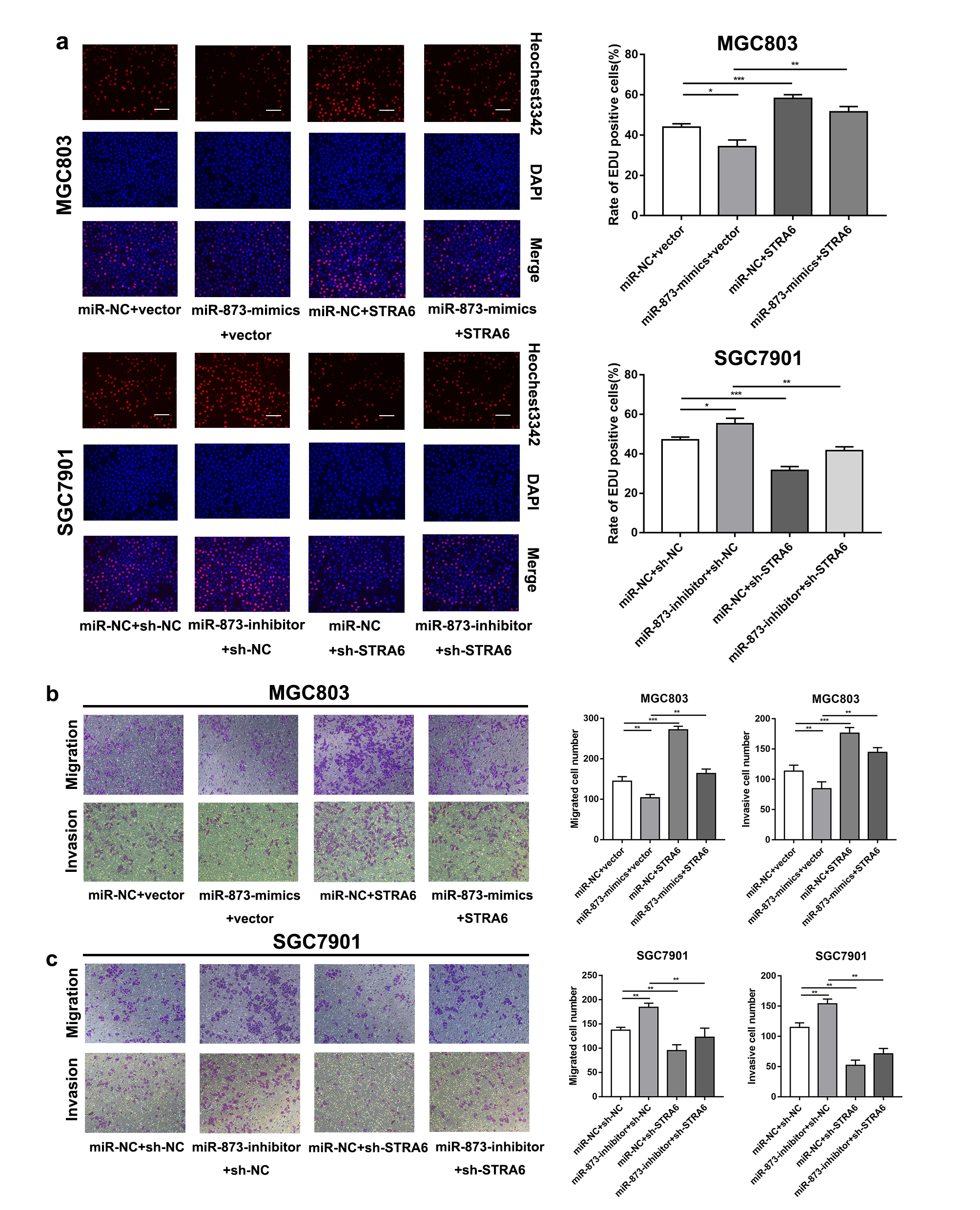

Supplement: Supplementary file 2 — Additional file 2: Figure S2. (a) EdU assays was conducted to examine the proliferation ability after co-transfecting with miR-NC, miR-873-mimics, vector or STRA6. (b) Transwell assay was used to analyze cell migration and invasion ability in each group. [file 13046_2019_1450_MOESM2_ESM.tif]

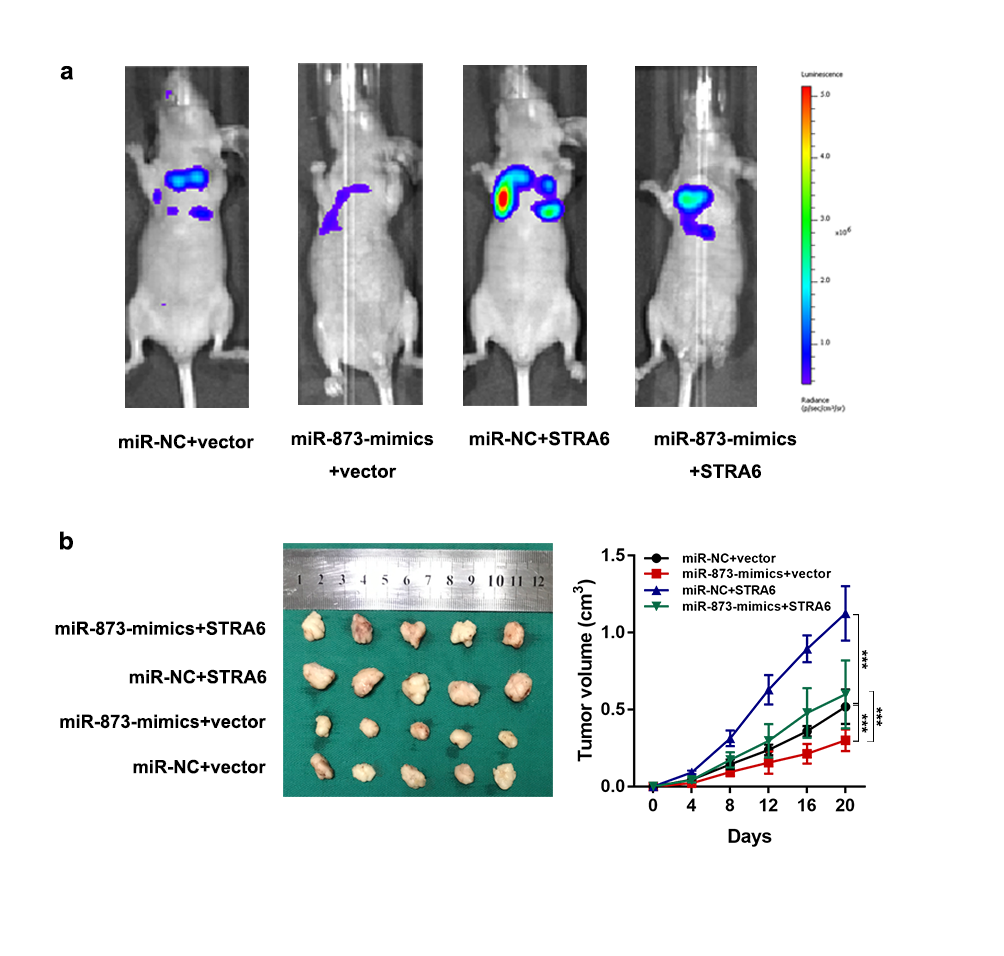

Supplement: Supplementary file 3 — Additional file 3: Figure S3. (a, b) The effect of miR-873-mimics on cell proliferation and metastasis were reversed by STRA6 overexpression in vivo. [file 13046_2019_1450_MOESM3_ESM.tif]

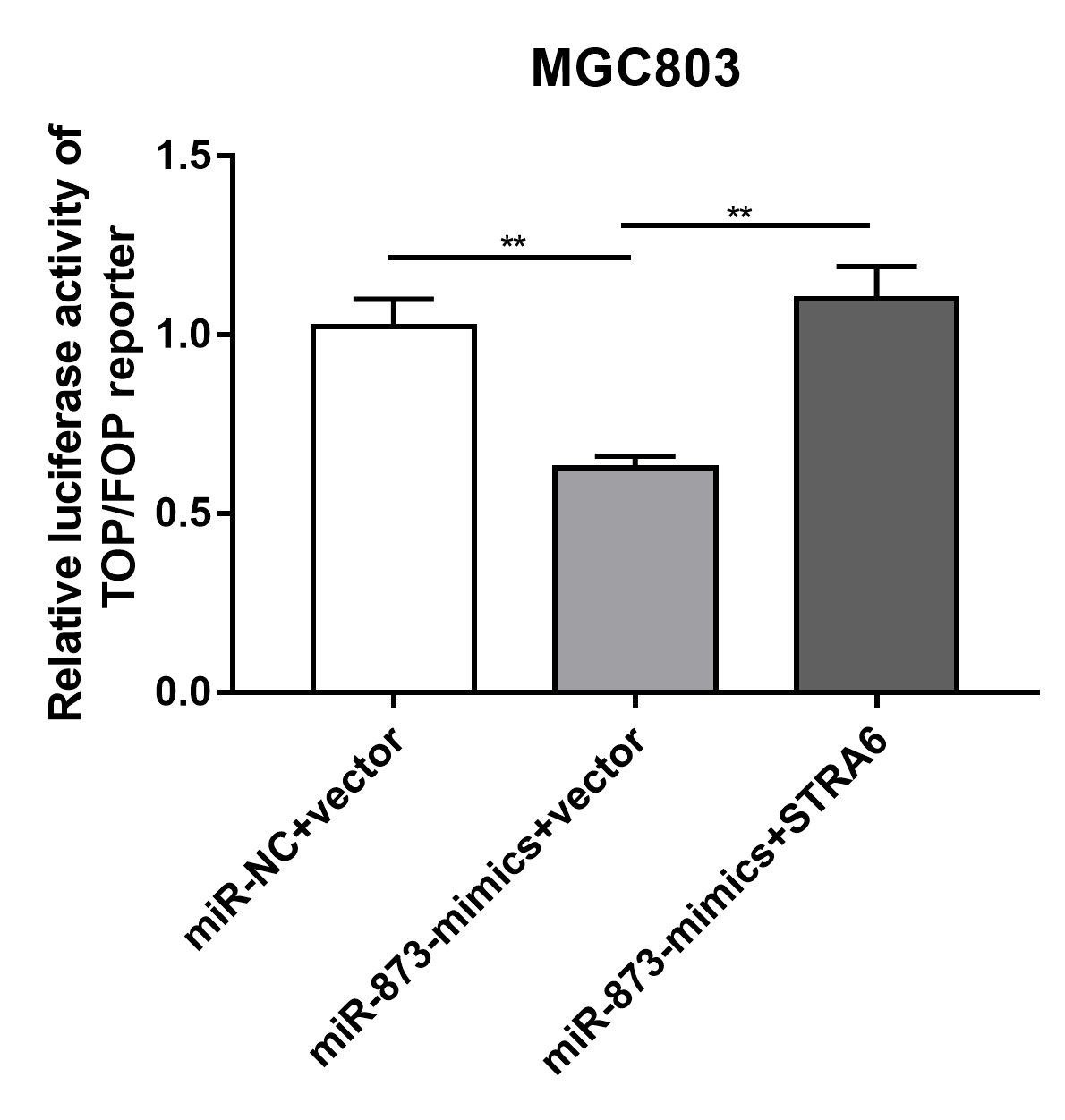

Supplement: Supplementary file 4 — Additional file 4: Figure S4. The TOP/FOP transcriptional activity was enhanced after up-regulating the expression of miR-873 and restoring STRA6 could partly reverse this effect. (TIF 200 kb) [file 13046_2019_1450_MOESM4_ESM.tif]
